# E-cigarette flavor and device preferences among US pregnant women who smoke: A latent class analysis

**DOI:** 10.18332/tpc/204745

**Published:** 2025-07-10

**Authors:** Emily A. Doherty, Kayleigh A. Gregory, Yu Lu, Page D. Dobbs

**Affiliations:** 1 Oklahoma State University Center for Health Sciences, Tulsa, United States; 2 Department of Family Science and Social Work, Miami University, Oxford, United States; 3 Department of Health and Exercise Science, University of Oklahoma, Norman, United States; 4 Department of Health, Human Performance and Recreation, College of Education and Health Professions, University of Arkansas, Fayetteville, United States

**Keywords:** pregnancy, e-cigarette, flavors, THC, dual use

## Abstract

**INTRODUCTION:**

Little is known about e-cigarette device and flavor preferences among pregnant women. The purpose of this study was to identify classes of e-cigarette use based on device and flavor preferences among pregnant women who report dual use of e-cigarettes and cigarettes.

**METHODS:**

A sample of pregnant women (n=118), aged 18–40 years, living in the US, with dual cigarette and e-cigarette use, completed a cross-sectional online survey. Participants reported e-cigarette characteristics including past 30-day e-cigarette device (cartridge-based, JUUL, tank, and disposable) and flavor use (tobacco, mint, spice, sweet, alcohol, combined), and use of e-cigarettes containing delta-9-tetrahydrocannabinol (THC) in pregnancy. We used latent class analysis to classify subgroups based on e-cigarette preferences in pregnancy and examined the association of sociodemographic variables and cigarette smoking frequency with class membership.

**RESULTS:**

We found four distinct classes of e-cigarette preferences: Class 1) tobacco, mint, and sweet JUUL (50.4%); Class 2) THC, all flavors, and JUUL (28.1%); Class 3) THC, all flavors, and all device (12.4%); and Class 4) THC, tobacco, mint, sweet, and tank device (9.1%). Pregnant women who smoked ≥11 cigarettes per day, compared to those who smoked 1–10 per day, were 5.22 (95% CI: 1.85–14.70) and 5.55 times (95% CI: 1.49–20.61) as likely to use THC, all flavors, and JUUL and all devices, respectively, compared with those who used tobacco, mint, and sweet flavors with JUUL.

**CONCLUSIONS:**

Pregnant dual users of cigarettes and e-cigarettes are a heterogenous group. Device and flavor differences should be considered when developing targeted messaging campaigns and prevention strategies.

## INTRODUCTION

As one of the leading attributers to neonatal morbidity and mortality, smoking during pregnancy can have multi-generational effects^1^. Given the established harm of smoking cigarettes, some women report using electronic cigarettes (e-cigarettes) while pregnant, because they believe them to be less harmful than cigarettes or as a cigarette cessation method^2^. However, research suggests there is insufficient evidence to support the use of e-cigarettes during pregnancy for this purpose^3^. Between 0.4% and 8% of pregnant women are estimated to use e-cigarettes during pregnancy^3^, with far higher rates of use among those who smoke cigarettes^4^. For instance, among a racially diverse low-income sample of pregnant women, 75% of those who smoked e-cigarettes reported concurrent cigarette use^5^.

Concerns of e-cigarette use among non-pregnant samples include immunosuppression, inflammation, increased risk of myocardial infarction and respiratory illness^6^. Evidence regarding an association between e-cigarette use in pregnancy and increased risk of adverse birth outcomes such as small for gestational age or low birth weight is mixed^7^. A recent study documented increased risk of fetal death among those who used mint or menthol e-cigarettes^7^. The extent of risk posed by e-cigarettes is further demonstrated by the 2019 outbreak of acute lung injury linked to the use of e-cigarettes containing the additive vitamin E acetate (e-cigarette and vaping related lung injury; EVALI), most commonly found in e-cigarettes with delta-9-tetrahydrocannabinol (THC; the primary psychoactive component of cannabis)^8^. During investigation of the EVALI outbreak, pregnant women in particular were warned to abstain from e-cigarette use for fear of deleterious health consequences^9^. E-cigarettes have evolved since their initial models that resembled cigarettes (ciga-like)^10^. They are now sold in a variety of shapes, sizes, and nicotine concentrations, including a wide range of flavors, and some contain THC. While product characteristics may impact product appeal, and therefore use, little research has investigated e-cigarette characteristic preferences among pregnant women^3^. Some pregnant women report e-cigarette use motivated by the availability of appealing flavors^11^. Use for appealing flavors nearly doubled in pregnancy compared with pre-pregnancy, suggesting flavors may be particularly appealing during this time^12^. Despite the suspected role of flavor appeal on e-cigarette use in pregnancy, to date a single study has examined flavor preferences (not exclusive to e-cigarettes) among pregnant women^13^. A sample of pregnant women (n=100) were found to prefer fruit and candy flavors more than flavors such as spice, coffee, and tobacco; however, this sample of 50% smokers included only nine women who reported e-cigarette use during pregnancy.

Despite the wide range of e-cigarette devices, there remains a gap in research regarding device preferences among pregnant populations. Device type may have implications for nicotine exposure, given nicotine salts found in cartridge-based devices make consumption of higher doses of nicotine more palatable^14^. Further, scant research has examined pregnant women’s use of e-cigarettes containing THC, despite concern for adverse effects^15^.

As has been recognized in the context of youth^16^, it is important to understand product characteristics that attract use among vulnerable populations at risk of nicotine exposure, such as pregnant women, to inform regulatory practices and prevention strategies. The present study aimed to identify distinct classes of e-cigarette users based on device type, flavors, and the use of THC in e-cigarettes among a sample of pregnant dual cigarette and e-cigarette users. Further, we sought to examine the association of sociodemographic and smoking characteristics with class membership. Given the high prevalence of e-cigarette use among pregnant women who smoke cigarettes and potential for heightened risks^17^, dual users represent an optimal sample for the present analysis.

## METHODS

### Participants

Participants were recruited from November to December 2019 via Dynata, a third-party paneling service, to complete a cross-sectional survey about their cigarette and e-cigarette use. Dynata offers prizes to panel participants in exchange for their participation in marketing and research questionnaires and specializes in hard-to-reach populations. Eligibility criteria included female sex assigned at birth, age 18–40 years, living in the United States, past 30-day use of cigarettes, and self-reported status of currently pregnant. Of the 3012 participants who initiated the survey, 267 met these eligibility criteria and were surveyed. For the current study, data were delimited to those who had used both cigarettes and e-cigarettes in the past 30 days (n=122). Due to incomplete responses (n=1) or non-use of one of the four e-cigarette devices analyzed in the current study (n=3), 118 participants remained in the final sample. All procedures were approved by the University of Oklahoma Institutional Review Board, and participants provided consent before starting the survey.

### Measures

Demographic measures included in the survey asked pregnant mothers about their age, race/ethnicity, and annual household income. To measure cigarette and e-cigarette use, participants were asked: ‘Have you used …’ with a list of substances (i.e. regular cigarettes, e-cigarettes, other tobacco products, alcohol, and marijuana). Response options included: ‘Yes, in the past 30 days’, ‘Yes, in the past year, but not in the past 30 days’, ‘Yes, but not in the past year’, and ‘No’. To measure smoking frequency, participants were asked: ‘In the past 30 days, on the days that you smoked cigarettes, about how many cigarettes did you smoke per day?’. Responses included: ‘Less than one cigarette per day’, ‘1 cigarette per day’, ‘2–5 cigarettes per day’, ‘6–10 cigarettes per day’, ‘11–20 cigarettes per day’, and ‘More than 20 cigarettes per day’.

To measure THC use, participants were asked: ‘During your pregnancy, have you put THC oil or liquid in your e-cigarette device (even once)?’. Responses included ‘yes’ (reported as e-cigarette with THC use) and ‘no’. To measure flavor preferences, participants were asked: ‘Which of the following flavors have you used when you used an e-cigarette and how recently did you use it?’. Informed by previous research, flavor options included: regular tobacco flavor, menthol or mint, clove or spice, candy, fruit, or chocolate, alcohol (wine or cognac), and combined flavors^13^. Response options included: ‘In the past 30 days’, ‘In the past year’, and ‘Tried, but not in the past year’, and ‘Never tried’. Participants were dichotomized into past 30-day use for each flavor (0 = did not use flavor in the past 30 days). To measure vaping devices used in the past 30 days, participants were asked to select all that applied to the question: ‘What type of e-cigarette device have you used during your pregnancy?’. Response options included popular devices at the time data were collected including: cartridge-based devices (SMOK, My Blu, STIG, etc.), JUUL, Tank/Mod devices, disposable e-cigarettes and some other device not listed here (with text box). At the time, JUUL brand devices had considerable market dominance^18^, and were therefore presented as a unique response category outside of cartridge-based devices. Use was categorized by use of each device during pregnancy (1) and lack of use during pregnancy (0).

### Data analysis

After reporting basic frequencies, we used Mplus 8.0 to conduct a latent class analysis (LCA)^19^. LCA was used to identify classes of pregnant mothers who smoked based on their e-cigarette flavor and device use patterns. Next, we sought to examine correlates with these classifications based on sociodemographic variables (age and income) and cigarettes smoked per day. LCA is a person-centered approach used to identify qualitatively different subgroups (classes) within a population based on responses to observed categorical items (indicator variables), such that participants with similar response patterns are grouped together. It has been used in prior research to classify groups of e-cigarette users, including based on reasons for wanting to quit, patterns of JUUL use behavior, and device and flavor preferences among young adult e-cigarette users^16,20^. For the current study, indicator variables included: one binary item about using e-cigarettes with THC, six binary items about flavor preferences, and four binary items about device preferences. To determine the model with the best fit, we examined the model fit indices and percent of the sample explained by individual classes, using the rule that the smallest class identified should be no less than 5% of the sample, in conjunction with model interpretability and conceptual meaningfullness^21^. Model indices included Log Likelihood (LL), Akaike information criterion (AIC),^22^ Bayesian Information Criterion (BIC), adjusted BIC, Entropy, and LoMendell Rubin Likelihood Ratio Test (LMR-LRT)^23^. For AIC, BIC, and adjusted BIC, better fit was determined using smaller values^24^. For the LMR-LRT, those with significant model fit improvement (alpha was set *a priori* to 0.05) for k-1 to k classes were determined to have better fit^24^. Entropy values closer to 1.00 indicated that participants are well grouped into their respective class^25^.

Next, we used a 3-step approach to identify covariates associated with identified classes^26,27^, as has been done in prior research^16^. By doing this, we were able to examine the relationship between sociodemographic information (age, race/ethnicity, income), cigarettes smoked per day, and classes of e-cigarette preference. After the best fitting latent class model was selected, participants were assigned to their most likely class based on posterior probabilities. A multinomial logistic regression model was used to regress latent class membership on covariates, adjusting for classification error. Adjusted odds ratios (AORs) and 95% confidence intervals (CIs) are reported. Confidence intervals not crossing 1 were considered statistically significant.

## RESULTS

### Descriptive analyses

As seen in Table 1, among the sample of included pregnant mothers that used e-cigarettes and cigarettes in the past 30 days (n=118), 44.1% were 31–40 years of age, 50.8% were Non-Hispanic White, and 33.9% reported a household income of less than $40000 per year. Thirty-three percent of the sample (n=39) smoked ≥11 cigarettes per day (at least half a pack). The most popular flavors used in the past 30 days included tobacco (69.5%), mint (61.9%), and fruit, candy, and chocolate (grouped into ‘sweet’; 61.0%). The most used device among this sample was JUUL, with 76.3% indicating that they had used this device during their pregnancy.

**Table 1:** Descriptive statistics from a cross-sectional survey of pregnant women with dual cigarette and e-cigarette use, 2019 (N=118)

*Characteristics*	*n (%)*
**Sociodemographic**	
**Age** (years)	
18–24	21 (17.8)
25–30	45 (38.1)
31–40	52 (44.1)
**Race/Ethnicity**	
Non-Hispanic White	60 (50.8)
Black/African American	24 (20.4)
Hispanic	13 (11.0)
Other races	21 (17.8)
**Income ($)**	
<20000	13 (11.0)
20000–39999	27 (22.9)
40000–59999	26 (22.0)
60000–99999	31 (26.3)
≥100000	21 (17.8)
**Smoking**	
**Cigarettes per day**	
1	7 (5.9)
2–5	40 (33.9)
6–10	32 (27.1)
11–20	28 (23.7)
>20	11 (9.3)
**E-cig flavors used in the past 30 days**	
Tobacco	82 (69.5)
Mint	73 (61.9)
Spice	39 (33.1)
Sweet	74 (61.0)
Alcohol	48 (40.7)
Combined	39 (33.1)
**E-cig devices used during pregnancy**	
Cartridge-based devices	49 (41.5)
JUUL	90 (76.3)
Tank device	34 (28.8)
Disposable devices	37 (31.4)
**Use of an e-cig containing THC during pregnancy**	
Yes	77 (65.3)

THC: delta-9-tetrahydrocannabinol. E-cig: electronic cigarette.

The next most popular device-types were cartridge-based (41.5%), disposable (31.4%), and tank e-cigarette devices (28.8%). A majority (52.1%, n=63) of the sample reported using one device type during pregnancy, 24.8% (n=30) used two, 10.7% (n=13) used three, and 9.9% (n=12) used all four device-types during pregnancy.

### Model selection

Models with 1- to 7-classes were tested. Exploration of models stopped at 7-classes because the smallest class size became smaller than 5%. Evaluating across all model fit indices, we found a 4-class model provided an optimal model. Although the BIC favored the 2-class model and the adjusted BIC was lowest for the 6-class model, the 4-class model had the smallest AIC. Further, the LMR-LRT for the 3-class and the 5-class (and subsequent-classes) model did not provide any better fit than the 4-class model. The 4-class model also had a high entropy of 0.95, indicating good classification. See Table 2 for model fit indices for 1- to 7-class solutions.

**Table 2:** Latent class model fit indices for 1- to 7-class solutions in a cross-sectional survey study of pregnant women with dual cigarette and e-cigarette use, 2019 (N=118)

*Class*	*LL*	*AIC*	*BIC*	*Adjusted BIC*	*Entropy*	*LMR-LRT*
1	-844.3	1710.6	1741.3	1706.5	-	-
2	-754.0	1554.0	1618.3	1545.6	0.86	177.5***
3	-733.0	1536.0	1633.8	1523.2	0.87	41.3
4	-714.2	1522.4	1653.8	1505.2	0.95	36.9*
5	-703.9	1525.8	1690.7	1504.2	0.95	20.3
6	-693.8	1529.7	1728.2	1503.7	0.95	21.3
7	-684.2	1534.4	1766.4	1504.0	0.98	20.7

LL: log likelihood. AIC: Akaike information criterion. BIC: Bayesian information criterion. Adjusted BIC: sample size adjusted Bayesian information criterion. LMR LRT: Lo-Mendell-Rubin likelihood ratio test. Lower AIC, BIC, and adjusted BIC values indicate better model fit. Higher entropy indicates better model classification. LMR-LRT compares models with k classes to a model with k-1 classes, significant p values indicate improvement in model fit with additional classes.

* p<0.05,

**p<0.01,

*** p<0.001.

### Identified classes

**Figure 1:**
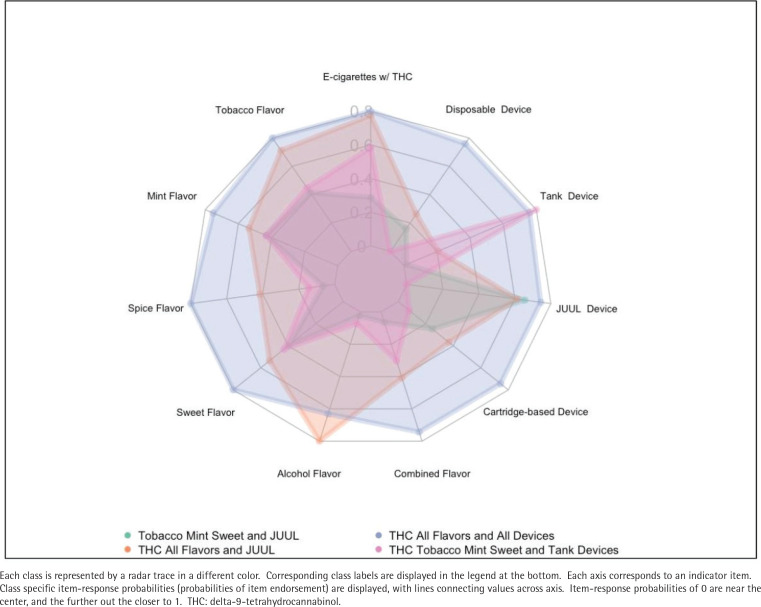
Rader plot of 4-class latent class model of pregnant women’s e-cigarette preferences, from a cross-sectional survey of pregnant women with dual cigarette and e-cigarette use, 2019 (N=118)

The largest class represented in our model was Tobacco, Mint, and Sweet JUUL with 50.4% of the sample. Pregnant mothers in this class reported moderate likelihood of using tobacco, mint, and sweet flavors with JUUL devices. The second most common class was THC, All Flavors, and JUUL (28.1%), where participants were likely to use THC in their e-cigarette, all flavors and JUUL devices. The third largest class was THC, All Flavors and All Devices (12.4%). This class had a moderate to high probability of indicating the use of all flavors, all devices, and e-cigarettes with THC. Lastly, THC, Tobacco, Mint, Sweet, and Tank Device (9.1%) made up the smallest class in our model. For this class, pregnant mothers indicated tank device use, with a moderate probability of using THC, tobacco flavor, mint flavor, and sweet flavor. See Table 3 for class specific item response probabilities in the 4-class model, depicted in Figure 1 as a radar plot^28^.

**Table 3:** Item-response probabilities from a 4-class model of e-cigarette preferences in a cross-sectional survey study of pregnant women with dual cigarette and e-cigarette use, 2019 (N=118)

	*Tobacco, Mint, Sweet, and JUUL*	*THC, All Flavors, and JUUL*	*THC, All Flavors, and All Devices*	*THC, Tobacco, Mint, Sweet, and Tank Devices*
**Class prevalence** (%)	50.4	28.1	12.4	9.1
**Items** (probability)				
**E-cigarettes with THC**	0.36	**0.97**	**1.00**	**0.73**
**E-cigarette flavor**				
Tobacco	**0.52**	**0.89**	**1.00**	**0.56**
Mint	**0.54**	**0.67**	**0.94**	**0.54**
Spice	0.08	**0.52**	**1.00**	0.18
Sweet	**0.50**	**0.67**	**1.00**	**0.54**
Alcohol	0.03	**1.00**	**0.79**	0.09
Combined	0.08	**0.51**	**0.93**	0.38
**Device**				
Cartridge-based	0.31	0.46	**0.93**	0.10
JUUL	**0.82**	**0.77**	**0.93**	0.00
Tank	0.02	0.26	**0.95**	**1.00**
Disposable	0.20	0.33	**0.95**	0.00

THC: delta-9-tetrahydrocannabinol. Boldface numbers represent moderate to high item-response probabilities (≥0.50). Example interpretation: 82% of pregnant women in the Tobacco, Mint, Sweet and JUUL class reported use of JUUL devices during pregnancy.

### Correlates of e-cigarette device and flavor classes

Using a multinomial logistic regression, covariates were examined to determine odds of class membership based on age, race/ethnicity, income, and number of cigarettes used per day on days smoked (Table 4). Compared to those who smoked 1–10 cigarettes per day, those who smoked ≥11 cigarettes per day were more likely to be in the THC, All Flavors, and JUUL class (AOR=5.22; 95% CI: 1.85–14.70) or in the THC, All Flavors, and All Devices class (AOR=5.55; 95% CI: 1.49–20.61) than in the Tobacco, Mint, Sweet, and JUUL class. Class membership, however, did not differ by age, race/ethnicity, or income.

**Table 4:** Multinomial logistic regression model results using Tobacco, Mint, Sweet and JUUL as the reference class, from a cross-sectional survey of pregnant women with dual cigarette and e-cigarette use, 2019 (N=118)

*Variables*	*THC, All Flavors, and JUUL* *AOR (95% CI)*	*THC, All Flavors, and All Devices* *AOR (95% CI)*	*THC, Tobacco, Mint, Sweet, and Tank Devices* *AOR (95% CI)*
**Age** (years) (ref. 18–30 years)			
31–40	0.46 (0.16–1.32)	2.53 (0.60–10.77)	4.32 (0.71–26.19)
**Race/ethnicity** (ref. White)			
All other races	1.26 (0.44–3.60)	1.65 (0.43–6.39)	1.72 (0.42–7.04)
**Income** ($) (ref. <39999)			
≥40000	1.32 (0.44–3.97)	0.39 (0.10–1.53)	2.88 (0.49–17.09)
**Cigarettes per day** (ref. 1–10)			
≥11	**5.22 (1.85–14.70)**	0.83 (0.14–4.86)	**5.55 (1.49–20.61)**

AOR: adjusted odds ratio; adjusting for the remaining variables in the table. Bolded values represent statistical significance (i.e. CI for OR does not cover 1).

## DISCUSSION

The current study advances research by identifying classes of pregnant mothers who smoke based on e-cigarette preferences (flavors, devices, and THC), demonstrating complex patterns of use. As of March 2025, four states (California, Massachusetts, New Jersey, New York) restricted the sales of all flavored e-cigarettes that have not received pre-market approval from the Food and Drug Administration (FDA). Most literature has been devoted to the impact of flavor availability on youth, given the epidemic proportions of e-cigarette use among adolescents; however, pregnant individuals may also be vulnerable to the appeal of e-cigarette flavors^3,13^ and may face harms associated with exposure to e-cigarette flavoring^7^. Tobacco, mint, and sweet flavors were preferred by 68.6%, 62% and 61.2% of our sample, respectively. Prior research found pregnant women (a small proportion of whom reported e-cigarette use in pregnancy) preferred sweet-flavored e-cigarettes (fruit and candy) and did not prefer tobacco flavored products^13^. In contrast, our findings suggest pregnant mothers used a wide variety of e-cigarette flavors, including tobacco flavor, with two of the four classes characterized by use of all flavors.

Debate surrounding e-cigarette flavor availability has centered around the risk of attracting novice nicotine users versus the ability to aid smoking cessation, with some suggesting that those who use e-cigarettes as a cessation tool are more successful with the availability of flavors^29^. However, little evidence supports this^3^. One-third of our sample reported smoking half a pack or more a day in the past 30 days, and greater cigarette use was associated with increased odds of being in the THC, all flavors and JUUL or the THC, all flavors and all devices classes, relative to the tobacco, mint, sweet and JUUL class. Thus, those who smoked more cigarettes per day appeared to be using a wider variety of e-cigarette flavors. Although frequency of e-cigarette use was not captured, this might equate to greater overall nicotine exposure among women in these classes^16^. Future studies should include measures of e-cigarette use frequency and nicotine concentration to provide overall estimates of nicotine exposure by e-cigarette preference.

E-cigarette devices vary in nicotine concentrations (e.g. 0–6%) and type of nicotine used (e.g. freebase vs nicotine salts). Among our sample, 76.3% endorsed the use of JUUL e-cigarettes during pregnancy and the two largest classes were characterized by JUUL use. These findings are consistent with JUUL’s market dominance at the time of data collection^30^. Further, JUUL has been noted for its appeal due to the ease of use, discreet and convenient nature, and availability of flavors, prior to October 2019^10^. Pregnant women report fear of being judged for using e-cigarettes or cigarettes, making a discreet option particularly appealing during this time^31^. JUUL and other cartridge-based devices have been noted for their high nicotine concentrations, with a JUUL pod found to deliver the nicotine equivalent of a pack of cigarettes^32^. Given known risks to the fetus from nicotine exposure^33^, use of high nicotine e-cigarette devices in pregnancy is alarming.

While those in the largest class (tobacco, mint, sweet and JUUL) had a low probability of THC use, all other classes had a moderate to high probability of using e-cigarettes with THC at least once during pregnancy. Similar to e-cigarettes, the effects of prenatal cannabis exposure are a growing area of research. Prenatal cannabis exposure has been linked to increased risk for low birth weight, preterm birth, and small for gestational age^34^. Further, little is known about the consequences of nicotine and THC combined. The moderate probability of using e-cigarettes with THC, which spans three out of four classes, is a major concern given this pattern of use may expose the fetus to various harmful chemicals (e.g. nicotine and THC).

A recent study suggested certain flavors (i.e. mint or menthol) are associated with higher risk of fetal death^7^; thus, conducting toxicity analyses of flavors preferred by pregnant women could offer an important contribution to educating pregnant individuals, healthcare professionals, and regulatory agencies. Understanding product preferences is important for regulatory agencies, such as the FDA Center for Tobacco Products (CTP) that can regulate product characterization. The identification of classes defined by indiscriminate device and flavor use, associated with greater cigarette consumption, mirrors findings seen in young adults^16^. Given the popularity of tobacco flavor across classes, restriction of sweet, flavored e-cigarettes may not be an effective deterrent of e-cigarette use among pregnant dual users. Use of JUUL, a higher nicotine device, defined the two largest classes of pregnant dual users. Therefore, restrictions on nicotine concentrations, such as those implemented in countries other than the United States^35^, may reduce some pregnant dual users’ nicotine consumption.

### Strengths and limitations

The current study is strengthened by assessing a variety of e-cigarette flavor preferences, as well as device type and use of e-cigarettes containing THC among pregnant women who engage in dual use. Furthermore, the research was conducted at a crucial time during the EVALI outbreak. Understanding the specific practices which led to these cases and guiding public health response are crucial in the prevention of recurring incidents in the future. These findings should be interpreted with the following limitations. First, e-cigarette use was measured by self-report and may be subject to social desirability bias or errors in recall. Second, given the fast-evolving e-cigarette market, device preferences may have changed since data were collected. Data collection took place immediately after JUUL suspended the sale of all flavors besides tobacco, mint, and menthol in anticipation of the FDA cartridge-based flavor restrictions announced January 2020. In the years following, sale of other flavored disposable e-cigarettes and high nicotine strength e-cigarettes have risen in the general population. Yet, the noteworthy gap in evidence of e-cigarette product preferences in pregnancy highlights the significance of the present study. Third, while we examined differences between e-cigarette preference classes by sociodemographic characteristics of age, race and ethnicity, and income, other variables may be associated with e-cigarette preferences during pregnancy including pregnancy trimester. Fourth, the present sample size was relatively small for LCA, for which more is considered better^36^. One issue which may be responsible for this, is failure to uncover classes with low class membership. Lastly, LCA is an exploratory approach, observed relationships therefore do not represent causality.

## CONCLUSIONS

With a changing regulatory landscape for e-cigarette flavor availability, it is important to understand device and flavor preferences among vulnerable populations, including pregnant women. Our findings suggest pregnant women who engage in dual use demonstrate diverse patterns of e-cigarette preferences, with a large group indicating use of high-nicotine devices. Among our sample, those who smoked more cigarettes per day were more likely to use a wide variety of e-cigarette flavors and THC, which raises concern for polysubstance exposure. Increased understanding of e-cigarette preference profiles can inform prevention efforts, provide insight for needed education, and inform effective regulatory actions to reduce use of tobacco and nicotine products among pregnant women. Future research examining how pregnant women’s e-cigarette preferences vary by e-cigarette use frequency, biomarkers of nicotine intake, and use motivations in the current landscape are warranted.

## Data Availability

The data supporting this research are available from the authors on reasonable request.
